# The octopamine receptor OAα1 influences oogenesis and reproductive performance in *Rhodnius prolixus*

**DOI:** 10.1371/journal.pone.0296463

**Published:** 2023-12-29

**Authors:** Luca Finetti, Ian Orchard, Angela B. Lange

**Affiliations:** Department of Biology, University of Toronto Mississauga, Mississauga, ON, Canada; Universidade Federal do Rio de Janeiro, BRAZIL

## Abstract

The control of reproductive processes in *Rhodnius prolixus* involves a variety of neuroactive chemicals. Among these, several studies have suggested that the biogenic amine octopamine (OA), might play an active role in these processes. Here, we investigate the molecular profile of the *R*. *prolixus* α adrenergic-like OA receptor 1 (RpOAα1-R) and its role in egg production. Comparative molecular analyses confirm that the RpOAα1-R gene codes for a true OAα1 receptor. The RpOAα1-R transcript is highly expressed in tissues associated with egg production, and after a blood meal, which is the stimulus for full egg production in *R*. *prolixus*, the *RpOAα1-R* transcript is upregulated in the ovaries and spermatheca. After RNAi-mediated *RpOAα1-R* knockdown, an ovarian phenotype characterized by slow egg development is observed. Furthermore, an altered egg phenotype has been characterized with eggs that are deformed. Interestingly, there is no evidence of disruption in vitellogenin (Vg) synthesis by the fat body or uptake by the oocytes. On the other hand, *RpOAα1-R* downregulation is correlated with defective choriogenesis in the eggs. These results provide critical information concerning the role of OAα1-R in oogenesis in *R*. *prolixus*.

## Introduction

*Rhodnius prolixus*, also known as the kissing bug, is one of the major vectors of the Chagas disease parasite *Trypanosoma cruzi* [1,2]. *R*. *prolixus* is an obligate blood feeder and requires nutrients from a blood meal to undergo growth, development, and reproduction. In the adult female, a blood meal triggers neuroendocrinological events that result in a cascade of intracellular pathways associated with egg development [2]. Among these, are pathways mediated by two hormones; juvenile hormone (JH) and 20-hydroxyecdysone (20E) [2–6]. However, other neurochemicals are also involved in *R*. *prolixus* reproductive processes, including biogenic amines [7–9].

Biogenic amines are evolutionarily conserved molecules that function in neuro-signaling and play crucial roles in several physiological functions. Most biogenic amines are common to both vertebrates and invertebrates (dopamine, serotonin, and histamine), while some others are preferentially synthesized in either vertebrates, such as epinephrine and norepinephrine, or in invertebrates, such as tyramine (TA) and octopamine (OA) [10]. OA is an important neurochemical in insects, synthesized from TA by the enzyme tyramine β-hydroxylase (TβH) [11]. OA acts as a neurotransmitter, neuromodulator, and neurohormone controlling several physiological and behavioral aspects in insects such as locomotion, courtship, memory, and reproduction [12–15]. OA exerts its effects through binding to specific G-protein coupled receptors (GPCRs), resulting in changes in second messenger levels, such as increasing intracellular Ca^2+^ or altering cAMP levels [14]. Based on their structure and intracellular pathway activated, OA receptors are classified into two α adrenergic-like OA receptors (OAα1-R, OAα2-R) and three β adrenergic-like OA receptors (OAβ1-R, OAβ2-R, OAβ3-R) [16].

OA signaling is well-known to be involved in insect reproduction [17]. Both OA and OA receptors control and modulate physiological processes directly related to oogenesis and oviposition. In several insect species, *R*. *prolixus* included, OA modulates the myogenic activity of a variety of reproductive muscles, including those associated with the ovaries, oviduct, and bursa. For example, OA reduces the amplitude and frequency of oviduct contractions in *Locusta migratoria* [18], *Drosophila melanogaster* [19], and *R*. *prolixus* [7]. In *R*. *prolixus* OA decreases spontaneous contractions of the oviducts and bursa and this action is mediated via increases in cAMP [7]. Interestingly, the absence of OA, caused by TβH knockdown, has been linked with sterility in *D*. *melanogaster* [20–22], a dramatic phenotype rescued by OA ingestion [23]. These physiological effects are linked to two OA receptors: OAα1-R (termed octopamine receptor in mushroom bodies, OAMB in *D*. *melanogaster*) and OAβ2-R. In *D*. *melanogaster*, the knockdown of OAMB causes female sterility due to egg retention [24,25]. Deady & Sun [26] found that OAMB is necessary for follicle rupture during egg development in *D*. *melanogaster*. Moreover, an OAMB-mediated intracellular pathway has been linked with the proliferation of the female germinal stem cells in *D*. *melanogaster* [27]. OAβ2-R is also needed for ovulation with a *D*. *melanogaster* OAβ2-R mutant being defective in egg-laying [28]. Egg retention in ovaries has also been observed in *Nilaparvata lugens* females lacking OAβ2-R [29]. The β-adrenergic receptor antagonist phentolamine blocks the inhibitory effects of OA on *R*. *prolixus* oviducts [7], and so taken together, OA may modulate oviduct musculature via OAβ2-R.

The involvement of OAα1-R in *R*. *prolixus* reproduction has not been reported, and so this paper examines the possible role of OAα1-R in egg production, particularly oogenesis, egg laying, and hatching in *R*. *prolixus*.

## Material and methods

### *R*. *prolixus* rearing

Adult females of *R*. *prolixus* were taken from an established colony at the University of Toronto Mississauga and reared in incubators at 25°C under high humidity (∼50%). Adult females at 7 days post-ecdysis (d PE) were fed through an artificial feeding membrane on defibrinated rabbit blood (Cedarlane Laboratories Inc., Burlington, ON, Canada) in order to stimulate normal egg production. Adult females post blood meal (PBM) were placed individually in a cage with two fed males for copulation.

### Isolation and cloning of full-length RpOAα1-R

Total RNA was extracted from the reproductive system of adult *R*. *prolixus* females using TRIzol™ Reagent according to the manufacturer’s protocol (Thermo Fisher Scientific, Waltham, Massachusetts, USA), quantified in a spectrophotometer DS-11+ (DeNovix Inc., Wilmington, DE, USA) and analyzed by 1% w/v agarose gel electrophoresis. One μg of RNA was treated with DNase I (Thermo Fisher Scientific) and used for the synthesis of cDNA, carried out with SMARTer RACE 3’/5’ Kit (Takara, Japan) according to the manufacturer’s instructions. To identify the full *R*. *prolixus* OAα1 open reading frame (ORF), a blast search on VectorBase was performed using *Nilaparvata lugens* (XM_039440532.1) and *Cimex lectularius* (XM_024229855.1) *OAα1-R* sequences as templates. Two specific reverse primers (**S1 Table**) were designed for the amplification of the 5′ cDNA end of the receptor (5′ Modified RACE) using SMARTer RACE 3’/5’ Kit (Takara, Japan). The final PCR product corresponding to the complete *R*. *prolixus* OAα1-R ORF was amplified by specific primers (**S1 Table**), gel purified, cloned into pJET 1.2/blunt vector (Thermo Fisher Scientific) and transformed into *Escherichia coli* XL-1 chemically competent cells. Positive clones were selected using LB broth agar plates with 100 μg ml^−1^ ampicillin. Plasmid was then extracted by EZ-10 spin column plasmid DNA miniprep kit (Bio Basic, Ontario, Canada) and verified by DNA sequencing (Macrogen, Seul, South Korea). The sequence, named *RpOAα1-R*, is deposited in GenBank with the accession number (OR248009).

### Bioinformatics

Phylogenetic maximum likelihood analysis (1000-fold bootstrap resampling) was performed using MEGA software. The *D*. *melanogaster* odorant receptor Orco (OR83b) was used as an outgroup to root the tree (NP_001097687.1). Multiple protein sequence alignments between the deduced amino acid sequence of *R*. *prolixus* OAα1-R and other biogenic amine receptor sequences were performed using Clustal Omega (https://www.ebi.ac.uk/Tools/msa/clustalo/) and BioEdit Sequence Alignment Editor 7.2.6.1. Molecular characterization of RpOAα1-R was performed by TMHMM v.2.0 Server (https://services.healthtech.dtu.dk/services/TMHMM-2.0/). N-glycosylation sites and targets for protein kinase A and protein kinase C phosphorylation were predicted by NetNGlyc 1.0 Server (https://services.healthtech.dtu.dk/service.php?NetNGlyc-1.0) and NetPhos 3.1 Server (https://services.healthtech.dtu.dk/service.php?NetPhos-3.1).

### RNA extraction and RT-qPCR analysis

The *RpOAα1-R* expression profile was investigated in the central nervous system (CNS), fat body, bursa, oviducts, spermatheca, tropharium, and follicles of 7 d post ecdysis (PE) unfed adult females. In addition, *RpOAα1-R* mRNA was analyzed throughout different time points PBM (1, 3, and 5 d) in the same tissues as above. For this purpose, 7 d PE adult *R*. *prolixus* females were fed with rabbit blood, and tissues collected starting from 1 d PBM. The individual tissues from three animals were dissected and pooled for each biological replicate. In order to investigate the expression profile of JH and 20E pathway actors, fat body and ovaries were dissected from 6 d PBM females, treated with dsOAα1-R or control dsRNA. Total RNA was extracted using TRIzol™ Reagent according to the manufacturer’s protocol (Thermo Fisher Scientific, Waltham, Massachusetts, USA). Five hundred ng of purified RNA was then treated with Turbo DNase I (Invitrogen, Waltham, Massachusetts, USA) and used for cDNA synthesis, carried out with High-Capacity cDNA Reverse Transcription kit (Applied Biosystem, Waltham, Massachusetts, USA) according to the manufacturer’s instructions. qPCR was performed using a 384-CFX Connect Real-Time PCR Detection System (Bio-Rad, Hercules, California, USA) in a 10 μL reaction mixture containing 4.2 μL of cDNA previously diluted 1:5, 5 μL of Advanced qPCR Master Mix (Wisent Bioproducts Inc., Saint-Jean-Baptiste, QC, Canada), 0.4 μL forward primer (10 μmol l^−1^), and 0.4 μL reverse primer (10 μmol l^−1^). Thermal cycling conditions were 95°C for 3 min, 40 cycles at 95°C for 15 s, and 60°C for 30 s. After the cycling protocol, a melting-curve analysis from 65 to 95°C was applied. mRNA abundance of the analyzed genes was normalized by the 2^-ΔΔCt^ method [30] or the relative quantification method [31] using *Actin* and *Rp49* [32] primers (**S1 Table**). Each biological sample was performed having two technical replicates and using no-template controls.

### Fluorescent in situ hybridization (FISH)

The fluorescent in situ hybridization (FISH) technique was used to determine the expression pattern of RpOAα1-R in the female reproductive system. The OAα1-R fragment amplification was performed by using antisense gene-specific primers containing the T7 RNA polymerase promoter sequence (**S1 Table**). Then, the DIG RNA labeling kit SP6/T7 (Roche Applied Science, Mannheim, Germany) was used in order to synthesize the digoxigenin (DIG)-labeled antisense RNA probes. Hybridization was done by incubating the reproductive system from unfed *R*. *prolixus* females overnight, at 4°C, with 100 ng (final concentration: 1 ng/μL) of the specific probe. The signal specificity of the antisense RNA probe was tested on insects treated with dsOAα1-R. Then, a biotin-SP-conjugated monoclonal mouse anti-digoxigenin antibody (Jackson ImmunoResearch Laboratories Inc., West Grove, PA, USA) was incubated (1:400) overnight at 4°C with the tissues, under agitation. The tissues were then washed and incubated in 1:100 horseradish peroxidase-streptavidin solution (Molecular Probes, Eugene, OR, USA). For signal amplification, tissues were incubated with Alexa Fluor 568 tyramide solution (Molecular Probes, Eugene, OR, USA). Finally, tissues were mounted on slides with one drop of Fluoroshield (Sigma-Aldrich, ON, Canada). A confocal microscope LSM-800 (Carl Zeiss, Jena, Germany) with the Zeiss LSM Image Browser software was used to obtain and analyse the pictures.

### Double-stranded RNA design and synthesis

BlasTaq™ 2X PCR Taq MasterMix (ABM, Vancouver, BC, Canada) and primers with 5’ extensions containing T7 promoters (**S1 Table**) were used to generate the *R*. *prolixus* dsRNA for RpOAα1-R and the control dsRNA (ampicillin resistance gene or ARG) amplicons, 350–500 bp long. Then, the in vitro dsRNA synthesis was performed by T7 RiboMAX™ Express Large Scale RNA Production System (PROMEGA, Madison, Wisconsin, USA). The RpOAα1-R sequence used to synthesize the dsRNA is shown in **S1 Fig**.

### Knockdown of transcript expression using double-stranded RNA

RpOAα1-R downregulation was achieved by injecting 7 d PE *R*. *prolixus* adult females with 5 μL (1 μg/μL) of specific dsRNA using a 10 μL Hamilton 80330 standard microliter syringe (Reno, Nevada, USA). The access to a blood meal was given 7 days after the injection. Since the amount of blood ingested affects the number of eggs laid, insects were weighed before and after the blood meal. The RNAi silencing efficiency was evaluated by RT-qPCR on the whole reproductive system from unfed insects 7 days PI, and 6 days PBM (13 days PI).

### Egg laying and hatching ratio analysis

The number of eggs laid by dsARG (control) and dsOAα1-injected *R*. *prolixus* adult females was recorded over 20 d PBM. Fifteen insects were used to monitor egg laying in each treatment. For the hatching ratio, eggs from control or dsOAα1-injected females were collected and 14 days after the first egg hatched the number of nymphs alive was recorded.

### Chorion protein extraction and SDS-PAGE

Fifteen eggs from dsOAα1-R (both phenotypes) and dsARG females were opened under a stereomicroscope in cold 0.01M Tris/HCl pH 8.4 to remove the yolk protein content. The chorions were then carefully washed, three times, in 0.01 M Tris/HCl pH 8.4. Then, the chorions were treated as previously described by Santos & Ramos [33]. Briefly, the chorions were homogenized in 8 M urea, 360 mM Tris/HCl (pH 8.4), and 30 mM dithiothreitol using a manual pestle and incubated at room temperature for 30 min. The samples were centrifuged at 12,000 g for 10 min, and the supernatant was collected. 15 μl of the urea-extracted proteins were loaded into a pre-made gel (percentage 4–20%, Mini-Protean TGX Stain-Free Precast Gels, Bio-Rad, Hercules, California, USA) and stained with Coomassie. Densitometry was performed using ImageJ.

### Statistical analysis

Graph Pad Prism 9.0 (La Jolla, California, USA) was used to process all the data presented in the article. All data represent means ± s.e.m., evaluated using one-way ANOVA followed by Dunnett’s test for multiple comparisons, or Student’s T test, or F test for comparing cumulative egg laying over time.

## Results

### Molecular characterization of RpOAα1-R

**Fig 1:**
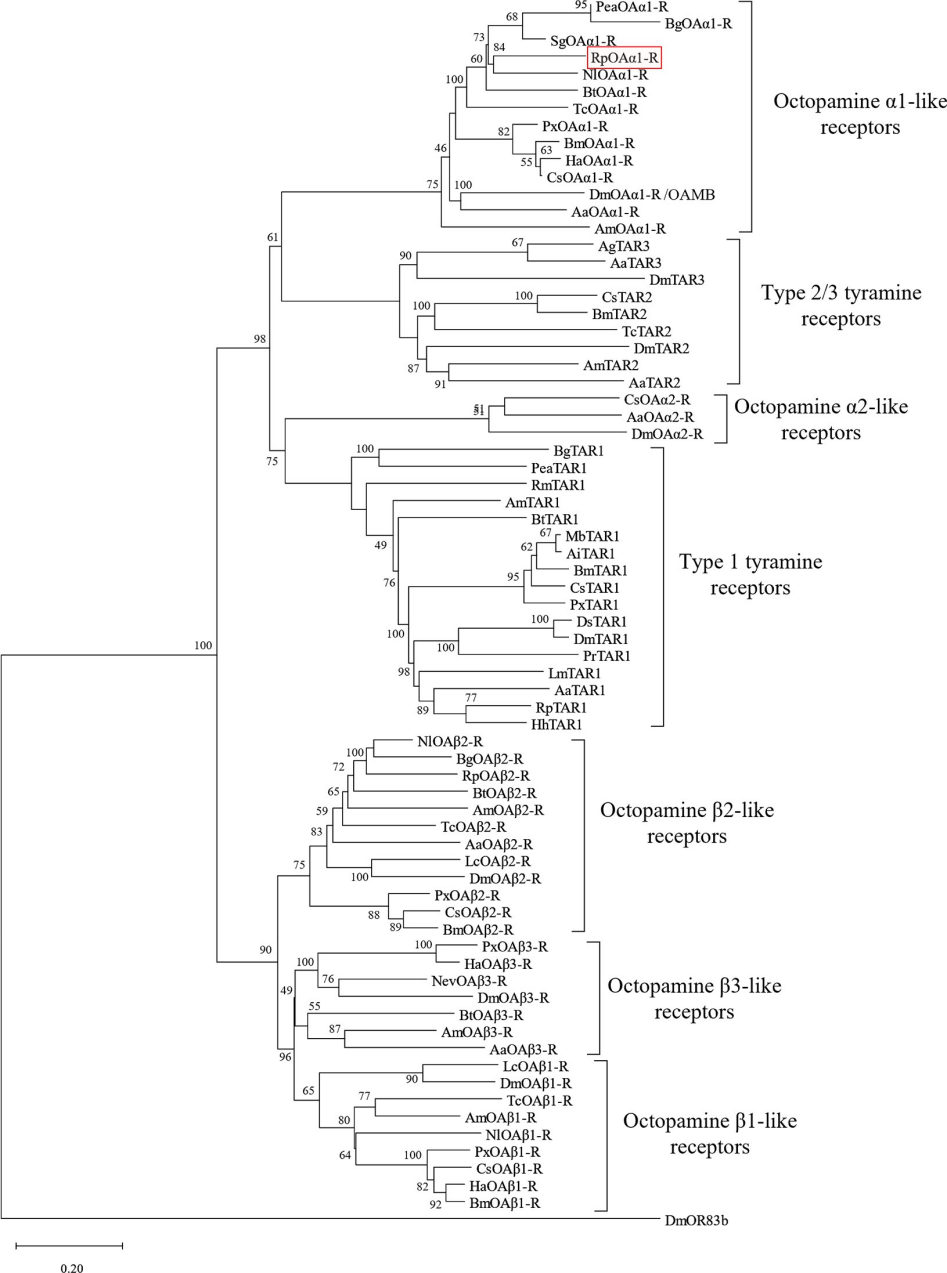
Phylogenetic relationship determined using maximum likelihood analysis of RpOAα1-R and other insect amine receptors. The characterized RpOAα1-R is outlined in red. The values shown at the nodes of the branches are the percentage bootstrap support (1000 replications) for each branch. Alignment was performed using the amino acid sequences found in GenBank (accession number for each sequence is noted and reported in **S3 Table**). *D*. *melanogaster* OR83b receptor was chosen as the outgroup.

The open reading frame sequence of RpOAα1-R is 1866 bp long and codes for a 621 amino acid polypeptide with a predicted MW of 68 kDa (**S2 Fig**). In terms of structural domains, TMHMM v.2.0 software suggests seven putative transmembrane domains, as expected for a GPCR (**S3 Fig**). The helixes are flanked by an extracellular N-terminus of 30 residues and a long intracellular C-terminus of 80 residues. Furthermore, the RpOAα1-R sequence contains a DRY conserved sequence just after transmembrane (TM) III, one N-glycosylation site in the extracellular N-terminus, and twelve P-glycosylation sites, two for PKA and ten for PKC (**S2 Table**). Moreover, at position 107 in TM III there is a conserved aspartic acid (D107) responsible for the interaction with OA and TA, the endogenous agonists of the receptor (Huang et al., 2007) (**S2 Fig**). The RpOAα1-R amino acid sequence was then compared with that of several OA and TA receptors, allowing the construction of a neighbor-joining phylogenetic tree using MEGA 11 (**Fig 1**). As expected, RpOAα1-R grouped with the OA α-adrenergic-like family and shares the highest percentage identity with the *Nilaparvata lugens* OA α-adrenergic-like receptor (GenBank accession no. XM_039440532.1), another Hemipteran. Based on the phylogenetic results, a multiple sequence alignment was performed between the RpOAα1-R amino acid sequence and other insect OAα1 receptors (**S4 Fig**). The analysis further confirms the similarity of RpOAα1-R to known OAα1 receptors, showing the typical GPCR structure with highly conserved domains corresponding to the transmembrane regions.

### *RpOAα1-R* is expressed in reproductive tissues in unfed and fed adult *R*. *prolixus* females

**Fig 2:**
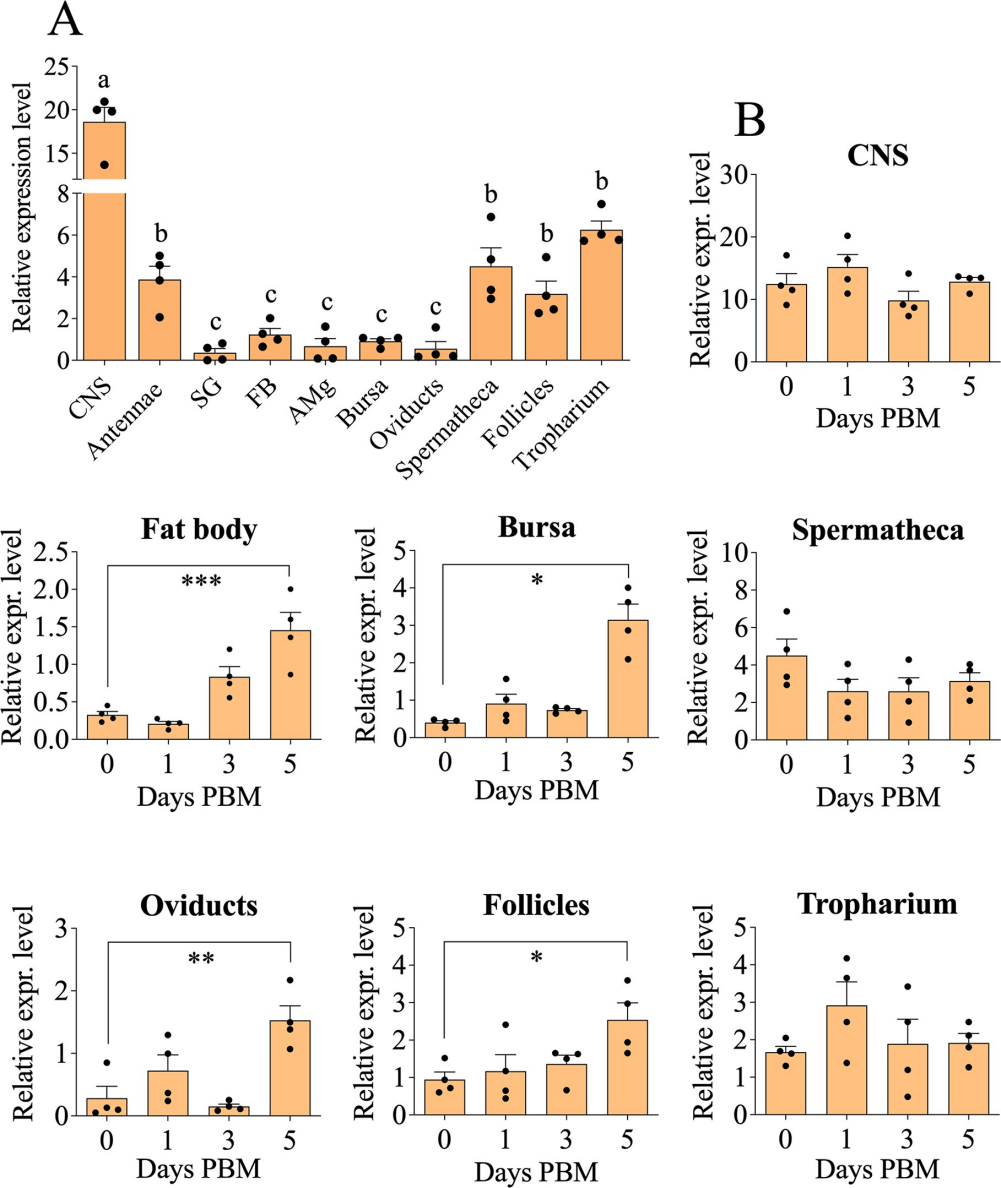
Tissue-specific expression profile of *RpOAα1-R* in adult *R*. *prolixus* females. Tissue transcript distribution of *RpOAα1-R* in unfed females of *R*. *prolixus* (**A**). Temporal expression of *RpOAα1-R* in different tissues 0 (unfed) to 5 d PBM in *R*. *prolixus* adult females: CNS, fat body, bursa, spermatheca, oviducts, follicles, and tropharium (**B**). Data represent means ± sem (n = 4 shown as individual data points, where each n represents a pool of tissues from 3 insects except for CNS where tissue is from 5 insects for each replicate). Statistical significance of p<0.05 is indicated by different letters in panel A. For panel B statistically significant compared to unfed conditions (0) was measured using a one-way ANOVA followed by multiple comparisons Bonferroni post hoc test. * p<0.05; ** p<0.01; *** p<0.001. CNS (central nervous system); SG (salivary glands); FB (fat body); AMg (anterior midgut); PBM (post blood meal).

To investigate the putative role of OAα1-R in *R*. *prolixus* reproduction, transcript expression of *RpOAα1-R* was quantified in *R*. *prolixus* tissues from both unfed and fed adult females (CNS, fat body, bursa, oviducts, spermatheca, follicles, and tropharium) (**Fig 2**). In unfed insects, *RpOAα1-R* is mostly expressed in the CNS and antennae, and in reproductive tissues such as the spermatheca and the ovariole structures, the follicles and tropharium (**Fig 2A**). After a blood meal, upregulation of *RpOAα1-R* expression is observed in the fat body, bursa, oviducts, and follicles, whereas transcript levels are unaffected in the CNS, spermatheca, and tropharium (**Fig 2B**).

### *RpOAα1-R* specific transcript expression in female *R*. *prolixus* reproductive tissues by fluorescent in situ hybridization

**Fig 3:**
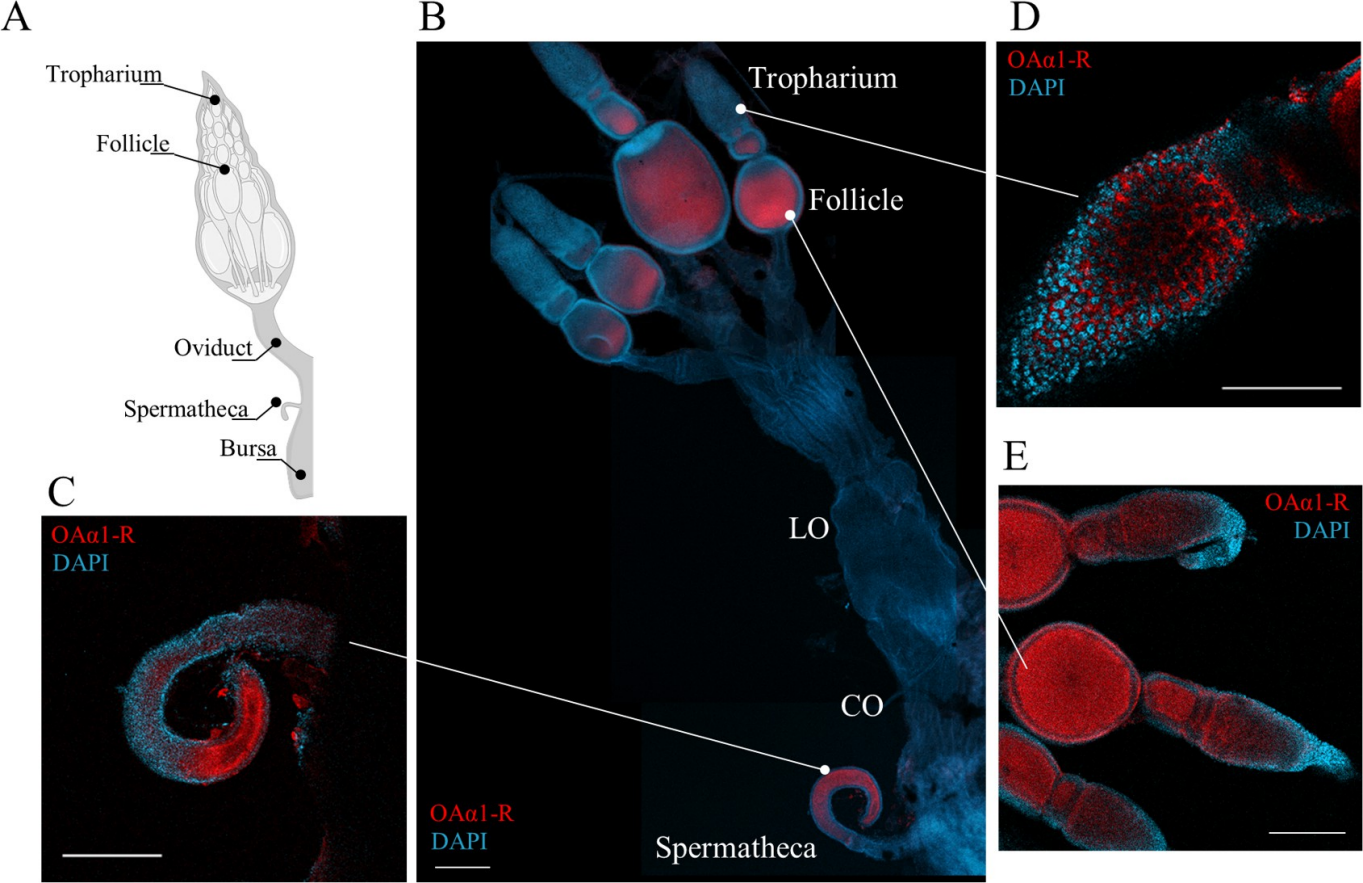
Fluorescent in situ hybridization analysis of *RpOAα1-R* transcript in the reproductive system of unfed *R*. *prolixus* adult females. Anatomy of the female *R*. *prolixus* reproductive system (**A**). Localization of the *RpOAα1-R* transcript across the reproductive system. CO: common oviduct; LO: lateral oviduct (**B**). Localization of the *RpOAα1-R* transcript in the spermatheca (**C**), tropharium (**D**), and follicle (**E**). FISH experiments were repeated twice, using 3 reproductive systems in each replicate.

To confirm the *RpOAα1-R* mRNA enrichment observed in the reproductive system by RT-qPCR, we examined the region-specific expression of the receptor in the reproductive system of unfed adult *R*. *prolixus* females by FISH (**Fig 3A**). Specific *RpOAα1-R* mRNA fluorescent signal is detected in the tropharium, follicles, and spermatheca, with a lower or no signal in the oviducts and bursa (**Fig 3B–3E**). Moreover, a specific signal for *RpOAα1-R* has been identified in the follicle cells and within the cytoplasm of the oocytes, as shown in the **Fig 3E**. This localization pattern reflects the expression profile of the *RpOAα1-R* transcript observed by RT-qPCR (**Fig 2A**). As a negative control, FISH experiments were performed on insects treated with dsOAα1-R to downregulate the receptor. As expected, lower or no labeling, specific for dsOAα1-R, is observed in either the spermatheca, tropharium or follicles (**S5 Fig**).

### RpOAα1-R signaling is essential for normal oogenesis

**Fig 4:**
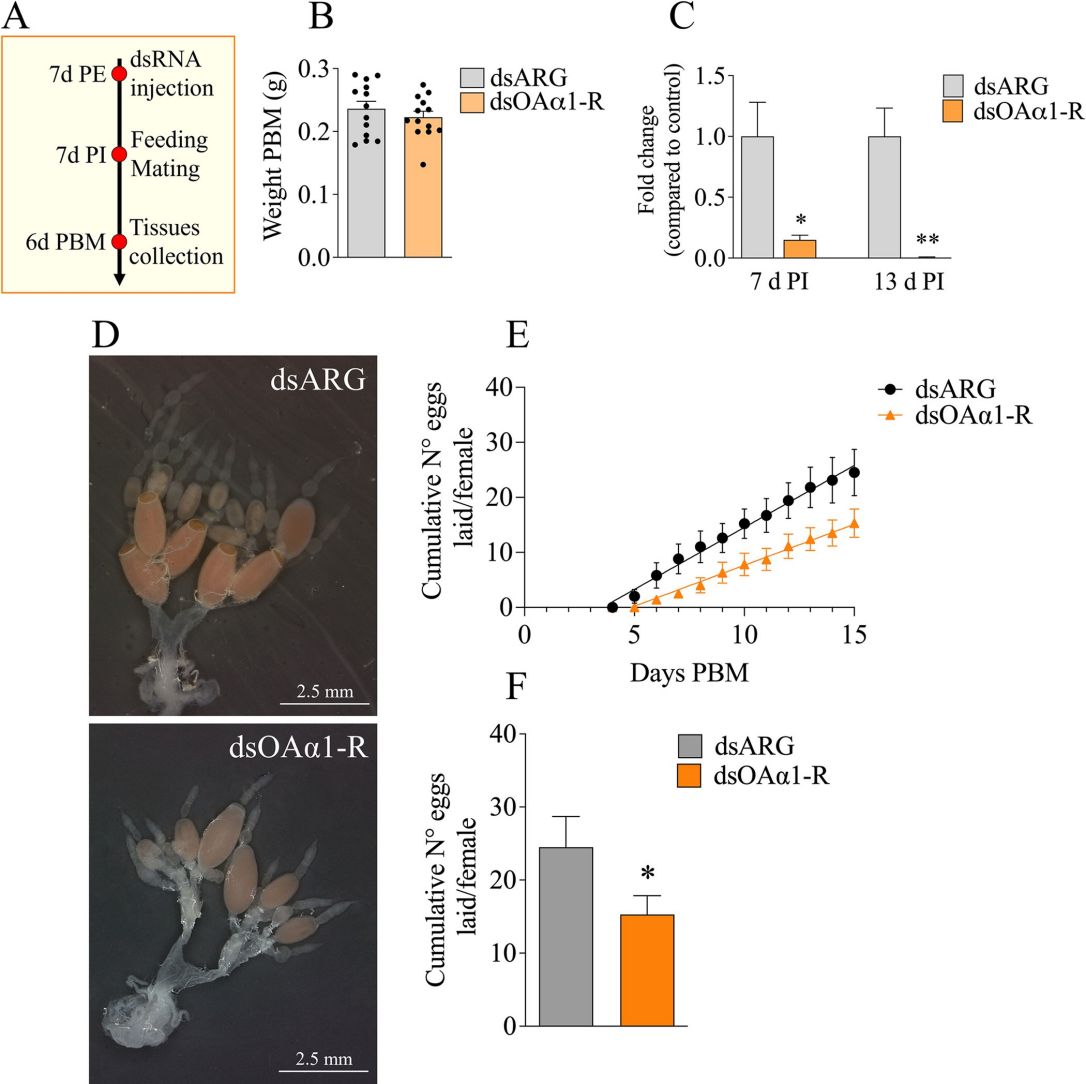
Effects of RNAi-mediated RpOAα1-R knockdown on *R*. *prolixus* oogenesis and egg laying. Experimental approach (**A**). Weight of dsRNA-injected insects following a blood meal. Data are the means ± sem of 13 independent replicates (each replicate composed of 1 female, and shown as individual data points) (**B**). The reproductive system was dissected 7 and 13 d post-injection (PI) to measure RNAi efficacy of *RpOAα1-R* transcript knockdown. The results are shown as the mean ± sem (n = 6, where each n represents tissue from 1 insect). Statistically significant differences were determined by Student’s t-test *p < 0.05; **p < 0.01 (**C**). The representative phenotype of the reproductive systems of adult *R*. *prolixus* females injected with dsARG or dsOAα1-R 13 d PI (equivalent to 6 d PBM) (**D**). Cumulative number of eggs laid per female injected with dsARG or dsOAα1-R over 15 d PBM. The results are shown as the mean ± sem (n = 15 insects). The statistical difference between linear regression slopes was performed by F-test (P = 0.0001) (**E**). Cumulative average of eggs laid per female by 15 d PBM. Statistical significance was determined by a Student’s t-test *p < 0.05 (**F**).

To investigate the role of RpOAα1-R in the reproductive physiology of *R*. *prolixus* adult females, dsRNA designed to specifically target the *RpOAα1-R* receptor sequence was injected into 7 d PE virgin adult females (**Fig 4A**) which were then fed and mated 7 d post-injection (PI) and tissue collected at 6 d PBM. To check for any potential effects of dsRNA injection on feeding (in terms of the amount of blood ingested), the injected insects were weighed before and after feeding; no significant differences between treated and control insects were observed (**Fig 4B**). The RNAi-mediated *RpOAα1-R* knockdown efficacy was investigated by RT-qPCR on full reproductive systems at 7 and 13 d PI (equivalent to 6 d PBM). The results show a significant reduction of the transcripts levels at both time points in the reproductive system (bursa, spermatheca, oviducts, and ovaries combined) (**Fig 4C**). RNAi-mediated *RpOAα1-R* knockdown results in ovaries with reduced egg development compared to the control (**Fig 4D**). Indeed, in the controls (dsARG), eggs are already chorionated and ovulated into the lateral oviducts at 6 d PBM (**Fig 4D)**, whereas this is less seen in the experimental insects. The reduced egg development in dsOAα1-R injected insects lead to a significant reduction in the cumulative number of eggs laid (**Fig 4E–4F**).

**Fig 5:**
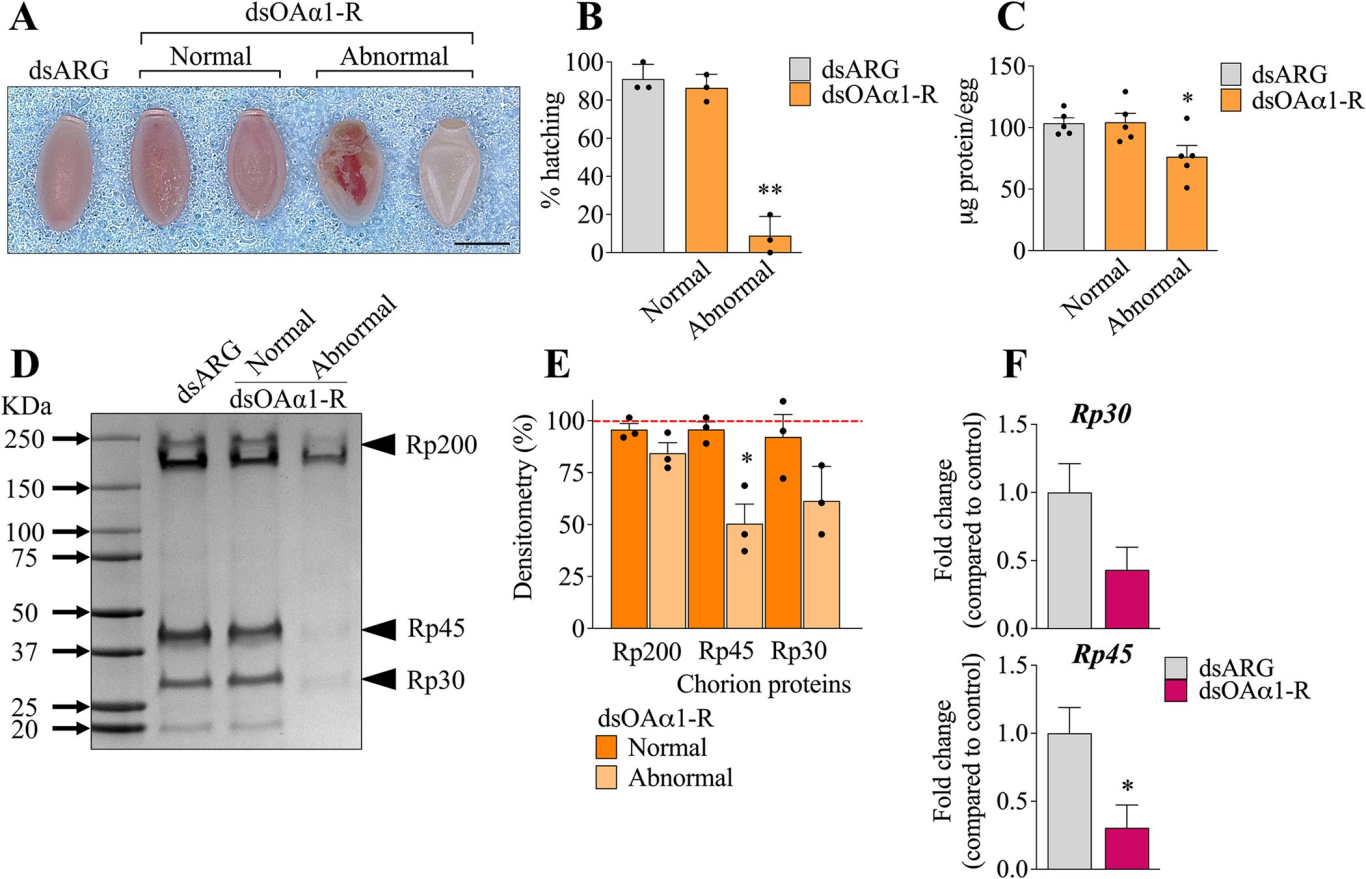
*RpOAα1-R* downregulation causes chorion-defects and unviable eggs. Representative images of dsARG and dsOAα1-R treated insect eggs. Scale bar: 1 mm (**A**). Hatching ratio throughout 14 days after the first egg hatched (n = 3, each n represents 15 eggs, shown as individual data points) (**B**). Total protein in eggs from *R*. *prolixus* females injected with dsARG or dsOAα1-R (n = 5 shown as individual data points, with each n composed of 3 eggs, 5 to 7 days after oviposition) (**C**). 4–20% SDS-PAGE of the urea-extracted proteins from control and dsOAα1-R egg chorions, collected 5 to 7 days after oviposition. Arrowheads indicate chorion proteins Rp30, Rp45, and Rp200 (**D**). The uncropped gel picture is available as **S6 Fig**. Densitometry analysis of the chorion proteins indicated in (**D**) performed using Image J. Graph shows mean ± sem (n = 3, shown as individual data points) (**E**). Effect of dsOAα1-R treatment on the chorion proteins *Rp30* and *Rp45* expression profile in the ovaries (n = 5). *p < 0.05 according to Student’s t-test (**F**).

In addition to the reduced rate of oviposition, the dsOAα1-R injected females lay eggs with two different phenotypes (**Fig 5A**). About 70% of the eggs laid from dsOAα1-R treated insects display a normal phenotype compared to controls (**Fig 5A**). The other 30% of the total eggs laid exhibit dramatic defects in their shape (**Fig 5A**). Moreover, only 10% of the eggs with abnormal phenotype are able to hatch, a dramatic reduction compared to the controls (**Fig 5B**). Both normal and abnormal eggs were laid throughout the 15d examined. Furthermore, the abnormal eggs have a reduced total protein content (**Fig 5C**). In addition to the altered hatching ratio and the lower protein content, abnormal eggs exhibit less amount of chorion proteins compared to the controls and to the normal eggs from dsOAα1-R injected females. Indeed, the chorion protein Rp45 is significantly reduced in the abnormal eggs laid from females with *RpOAα1-R* downregulated (**Fig 5E and 5F**). Furthermore, the transcripts for Rp30 and Rp45 are decreased in the ovaries from insects treated with dsOAα1-R, with *Rp45* statistically downregulated, suggesting a defective synthesis and/or accumulation of the chorion proteins compared to the controls (**Fig 5F**).

### Juvenile hormone (JH) and 20-hydroxyecdysone (20E) pathways are not involved in alterations in oogenesis induced by dsRpOAα1-R

**Fig 6:**
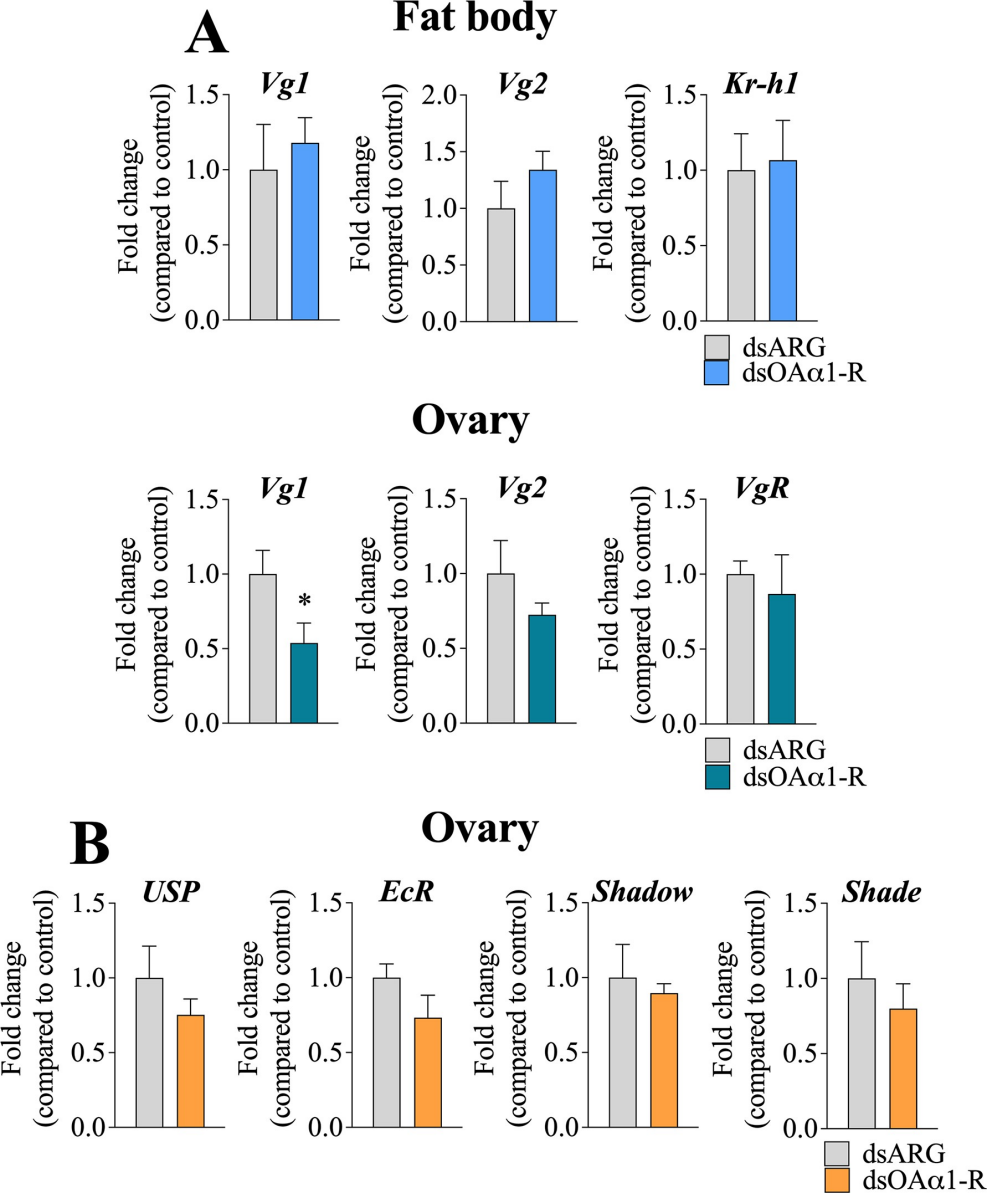
RT-qPCR analysis of JH and 20E pathway elements in the fat body and ovary of adult, 6 d PBM, mated *R*. *prolixus* females after dsOAα1-R treatment. RNAs were isolated from the fat body and ovary of dsARG (control) or dsOAα1-R injected *R*. *prolixus* females. RT-qPCR of mRNA expression of JH pathway elements *Vg1*, *Vg2*, and *Kr-h1* in the fat body, and *Vg1*, *Vg2*, and *VgR* in the ovaries (**A**). RT-qPCR of mRNA expression of 20E pathway elements *USP*, *EcR*, *Shadow*, and *Shade* in the ovaries (**B**). *Actin* and *Rp49* were used as reference genes. Data are mean ± sem of 5 independent replicates (each composed of tissues from 1 insect). Statistical analysis was performed using Student’s t-test. *p < 0.05.

Since JH signaling is directly involved in *R*. *prolixus* yolk protein synthesis [2,34] and 20E pathways have been recently correlated with *R*. *prolixus* oogenesis [6,35,36], their possible involvement in the altered egg phenotype after dsOAα1-R injection was investigated by RT-qPCR. To address this, the expression profile of proteins transcribed after the JH pathway is activated (vitellogenin genes *Vg1* and *Vg2*, *Krüppel homolog1 Kr-h1* and the vitellogenin receptor *VgR*) and the 20E pathway is activated (ultraspiracle protein *USP*, ecdysone receptor *EcR* and the Halloween genes *Shadow* and *Shade*) were explored. After dsOAα1-R injection, *Kr-h1* and *Vg1* and *Vg2* in the fat body at 6 d PBM were not altered but *Vg1* was downregulated by ~50% in the ovaries at 6 d PBM with no change in *Vg2* and *VgR* (**Fig 6A**). Concerning the 20E pathway, *USP*, *EcR* and the Halloween genes *Shade* and *Shadow* are not altered at in their expression profile at 6 d PBM compared to the controls (**Fig 6B**). Taken together, these data suggest that the effects of RNAi-mediated *RpOAα1-R* silencing do not involve JH or 20E in *R*. *prolixus*.

## Discussion

Although localized in tropical areas, climate change is responsible for creating new territories where *R*. *prolixus* can become endemic [37]. Moreover, *R*. *prolixus* is difficult to control by chemical approaches and the only way to treat Chagas disease is with two drugs that have limitations on treatment timing and serious side effects [38]. Because of its reduced susceptibility to traditional control strategies, new methods for *R*. *prolixus* control are needed. Therefore, identifying novel chemical compounds and new biological targets based on *R*. *prolixus* physiology is crucial. This study deals with the molecular characterization of the *R*. *prolixus* OAα1 receptor (RpOAα1-R). Moreover, by applying RNAi-mediated knockdown of RpOAα1-R, it was possible to reveal the importance of the OA pathway in the reproductive process in *R*. *prolixus*.

The RpOAα1-R polypeptide shares many structural features with OAα1 receptors from other insects and GPCRs in general [39]. RpOAα1-R contains seven highly conserved transmembrane segments, as expected for a GPCR, as well as phosphorylation and glycosylation sites, typical for this receptor class and essential for correct protein folding and receptor signaling [40,41]. Concerning the OA binding site, the main amino acid residue interacting with the endogenous agonist is an aspartic acid (Asp) located in TM III and well conserved in all the GPCRs [42]. The artificial mutation of the Asp residue in *Anopheles gambiae* AgOAR45B mutant resulted in the loss of responsiveness to OA [43]. Moreover, the mutant *Bombyx mori* OAα1-R D103A was unable to bind OA, indicating that the Asp residue is implicated in the binding and activation of the receptor by OA [44]. Therefore, it is mostly likely that the Asp residue localized in position 103 (D103) in RpOAα1-R polypeptide sequence may be implicated in OA binding. However, the same two studies showed that different residues localized in TM V and VI might also be involved in binding with OA, possibly revealing a more complex ligand/receptor-coupling mechanism [43,44].

To further investigate the role that RpOAα1-R might play in *R*. *prolixus* reproduction, an expression profile analysis of the receptor was performed. RT-qPCR on unfed *R*. *prolixus* adult females revealed that *RpOAα1-R* mRNA is enriched especially in the CNS compared to the other tissues analyzed. Since OA is one of the most important neurotransmitters in invertebrates, it is not surprising that the *RpOAα1-R* transcript is enriched in the CNS [14]. This is in keeping with that observed in other insects, including *Aedes aegypti*, *A*. *gambiae*, *D*. *melanogaster*, *Laodelphax striatellus*, and *Apis mellifera* [43,45–49]. Perhaps not surprisingly, *RpOAα1-R* levels are higher in the antennae of unfed *R*. *prolixus* females, probably given its well-established role in insect olfaction [50]. In line with this hypothesis, in *A*. *mellifera* and *D*. *melanogaster*, OAα1-R is found highly expressed in the antennal lobes, strongly suggesting a role in modulating olfactory responses [51]. Antennae are organs also responsible for sound perception, since OA has an important role in modulating sound responses in other blood-feeding insects (i.e., *A*. *gambiae*; [52–54]). Therefore, it might be possible that the enrichment of the *RpOAα1-R* transcript in the antennae reflects its possible role in hearing in *R*. *prolixus*.

In addition to the CNS and antennae, *RpOAα1-R* mRNA is enriched in the reproductive system, including the spermatheca, follicles, and tropharium; the latter two structures being part of the ovarioles of *R*. *prolixus* [2]. Interestingly the *RpOAα1-R* mRNA is not upregulated after a blood meal in the spermathecal or tropharium but is so in the follicles. OA may play a role in sperm storage/release in the spermatheca and maintenance in the tropharium that contains germ cells and nurse cells. The same receptor is important for reproductive success in other insects, including *D*. *melanogaster* and *L*. *striatellus* [27,49,55]. In *D*. *melanogaster*, OAMB modulates contraction of the oviduct and bursa after OA activation [22,26,56]. Furthermore, OAα1-R controls ovulation by modulating follicle rupture and consequently the release of the egg [55]. After a blood meal, *RpOAα1-R* expression levels are significantly increased in the fat body, bursa, oviduct, and follicles strongly suggesting that the receptor may play an important role in *R*. *prolixus* reproduction. This was confirmed by RNAi-mediated *RpOAα1-R* knockdown which significantly affects *R*. *prolixus* oogenesis, egg laying, and hatching.

Interestingly, the increase in *RpOAα1-R* expression after a blood meal is not immediate, but in most tissues, such as the oviduct and bursa, occurs 5 d PBM. We cannot exclude the possibility that the “late” increase in *RpOAα1-R* expression in the bursa and oviducts might be linked to the receptor’s role in muscle modulation, and expression in the follicles to ovulation. However, after *RpOAα1-R* downregulation, we did not observe any specific phenotypes, such as egg retention in the ovaries, oviduct or bursa and further experiments need to be performed in order to test these hypotheses.

Compared to the control group, fewer eggs are laid by *R*. *prolixus* dsOAα1-R-treated females. Moreover, 30% of the eggs laid are deformed resulting in hatching failure. In *A*. *gambiae*, the pharmacological alteration of the OAα1-R signaling by different OA agonist and/or antagonist clonidine, prazosin, and phenylephrine injection, affected egg development and egg laying [57]. Egg deformation is observed also in *L*. *striatellus* after *OAα1-R* silencing by RNAi [49]. In this case, the authors suggested that the altered egg development was due to an inhibited Vg synthesis occurring in the fat body upon *OAα1-R* downregulation. In our experiments, however, knockdown of *RpOAα1-R* did not alter JH or 20E pathways and, consequently, the synthesis of Vg in the fat body was comparable to that observed in the control group. Therefore, it is likely that the transcriptional increase of RpOAα1-R in the fat body PBM is not attributable to a direct role of the receptor in modulating Vg synthesis in the fat body but could be related to other nutrients or homeostatic processes. Furthermore, the expression levels of *VgR* in the ovaries, in both treated and control groups, were similar, indicating that no impairment in Vg uptake by ovaries occurred after *RpOAα1-R* downregulation. *Vg1* synthesis appeared altered only in the ovaries after dsOAα1-R injection. However, it remains to be investigated whether the *Vg1* transcript downregulation in the ovaries is directly affected by the RNAi-mediated *RpOAα1-R* downregulation or to a lesser protein demand from the defective eggs. Halloween genes essential for ecdysteroid synthesis, such as *shadow* and *shade*, play an important role in *R*. *prolixus* oogenesis [6,35], and there are profound effects on egg production and egg shape when ecdysteroid signaling is disrupted. However, after RNAi-mediated silencing of *RpOAα1-R*, the transcript expression levels of *shadow and shade* and the *USP* and *EcR* subunits were comparable between control and treated insect, suggesting that knockdown of *RpOAα1-R* did not alter expression of ecdysteroids.

A key step in oogenesis is the formation of the chorion, a process known as “choriogenesis”. During this process, the mature oocyte is coated with newly synthesized layers of eggshell, which are assembled and cover the entire oocyte surface. After choriogenesis, the eggs are ready to be fertilized and laid onto a substrate in the environment [58]. In *R*. *prolixus*, chorion formation is a complex multi-step process that requires different intracellular pathways, including autophagy [33,59]. Investigating the chorion properties in dsOAα1-R-treated *R*. *prolixus*, we observed alterations in both transcript and protein content of two specific chorion protein, Rp30 and Rp45. In *R*. *prolixus*, both proteins are expressed during the final stage of vitellogenesis, by the follicular cells [60]. Since follicles, during vitellogenesis, express higher transcript levels of *RpOAα1-R*, we cannot rule out the possibility of a direct OA-mediated pathway that modulates Rp30 and Rp45 synthesis during vitellogenesis in *R*. *prolixus*. However, since the cellular mechanisms that regulates Rp30 and Rp45 synthesis are not well characterized, this remains to be seen. It has recently been shown that the RNAi-mediated alteration of the ecdysone pathway in *R*. *prolixus* dramatically affected the synthesis of both Rp30 and Rp45 chorion proteins, producing abnormal eggs, as well as a reduction in number of eggs made and laid and their reduced hatching rate [6,35]. It is therefore possible that OA, through the RpOAα1-R receptor, is also involved in chorion synthesis during oogenesis but appears to act independent of 20E and JH.

## Conclusions

In conclusion, this study provides new insights on the molecular characterization and physiological function of the *R*. *prolixus* OAα1-R. Given its crucial role in *R*. *prolixus* reproduction, it could be considered as a new target for *R*. *prolixus* control. Further characterization of the OAα1 receptor, e.g. its pharmacological profile, is crucial to discover innovative control strategies targeting this octopamine receptor.

## Supporting information

S1 Fig *R*. *prolixus* OAα1-R sequence: regions chosen for dsRNA are highlighted. (DOCX)

S2 Fig *R*. *prolixus* OAα1-R sequence (accession number OR248009). (DOCX)

S3 Fig Hydropathy profile of the predicted RpOAα1-R amino acid sequence. (DOCX)

S4 Fig Amino acid sequence alignment of RpOAα1-R with orthologous receptors from *Nivaparlata lugens* (NlOAα1-R), *B*. *mori* (BmOAα1-R) and *Periplaneta americana* (PeaOAα1-R). (DOCX)

S5 Fig Controls for fluorescent in situ hybridization of RpOAα1-R transcript in the reproductive system of *R*. *prolixus* adult females. (DOCX)

S6 Fig The uncropped pictures for chorion protein gel shown in Figs 5, panel D. (DOCX)

S1 Table Primers used in this research work. (DOCX)

S2 Table Molecular features of *R*. *prolixus* OAα1-R. (DOCX)

S3 Table The accession number of OA and TA receptors from different insect species was used for the maximum likelihood phylogenetic analysis. (DOCX)

## Abbreviations

20E: 20-hydroxyecdysone
ARG: ampicillin resistance gene
CNS: central nervous system
d PBM: days post blood meal
d PE: days post-ecdysis
d PI: days post injection
dsRNA: double-stranded RNA
FB: fat body
GPCR: G protein-coupled receptor
JH: juvenile hormone
OA: octopamine
OAα1-R: α1 adrenergic-like OA receptors
RNAi: RNA interference
TA: tyramine
Vg: vitellogenin
