# Supplementary material for: The octopamine receptor OAα1 influences oogenesis and reproductive performance in Rhodnius prolixus
Source: PLoS One. 2023 Dec 29;18(12):e0296463. doi: 10.1371/journal.pone.0296463 (PMC10756544; doi:10.1371/journal.pone.0296463)
Supplement: S1 Fig — (DOCX) [file pone.0296463.s001.docx]

**Supplementary Figure S2.** *R. prolixus* OAα1-R sequence (accession number OR248009)

**
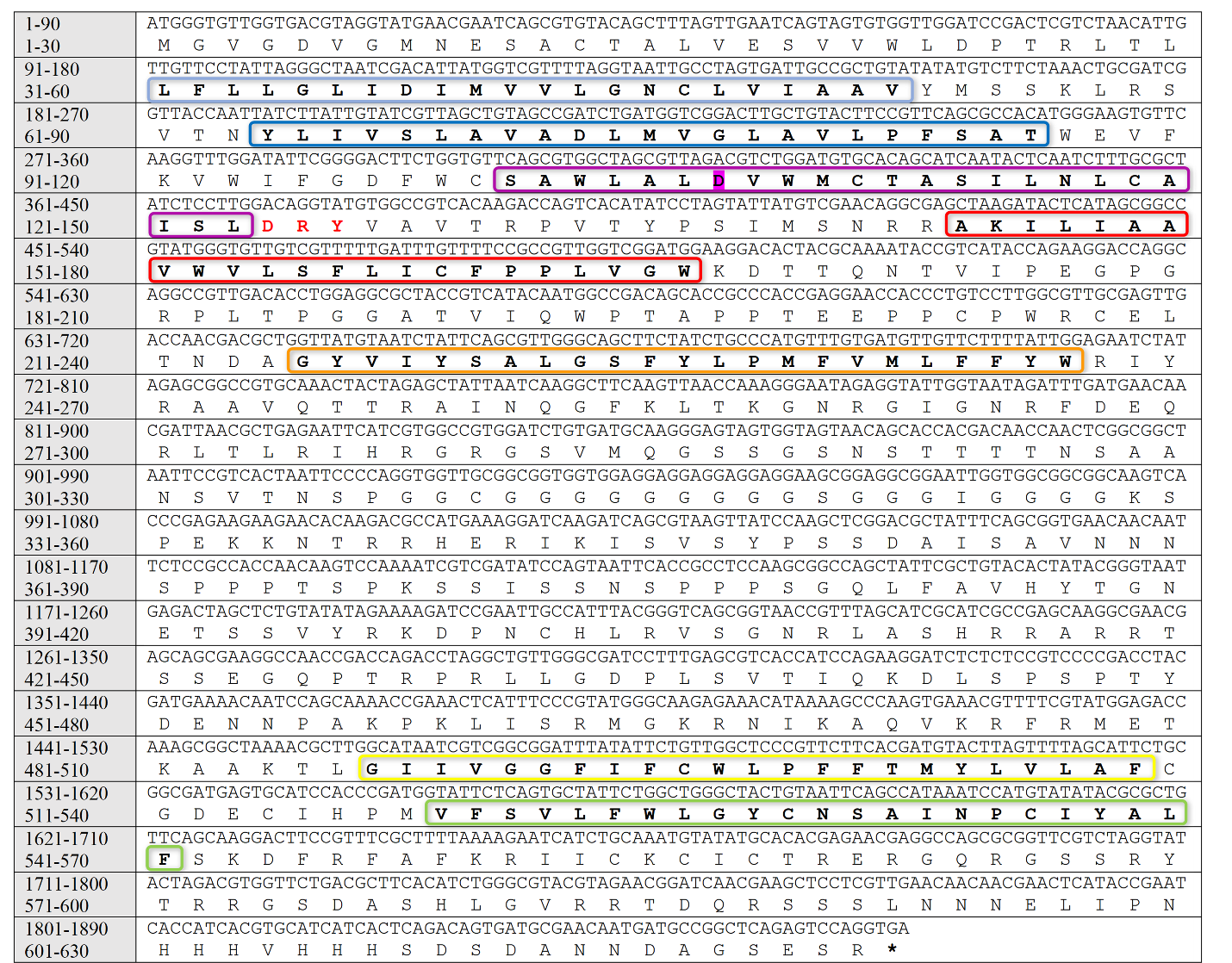
**

**Supplementary Figure S2.** **Nucleotide sequence of RpOAα1-R open reading frame and deduced amino acid sequence.** Prediction of the RpOAα1-R transmembrane segments (outlined) was obtained with TMHMM v. 2.0 software (**Supplementary Figure 3**). After the third transmembrane domain there is the DRY motif (highlighted in red) important for the stabilization of GPCRs between inactive and activate conformation. The aspartic acid in TM3 (D107), highly conserved in the α-adrenergic-like OA receptor family, is highlighted in a purple box.
