# Supplementary material for: The octopamine receptor OAα1 influences oogenesis and reproductive performance in Rhodnius prolixus
Source: PLoS One. 2023 Dec 29;18(12):e0296463. doi: 10.1371/journal.pone.0296463 (PMC10756544; doi:10.1371/journal.pone.0296463)
Supplement: S2 Fig — (DOCX) [file pone.0296463.s002.docx]

**
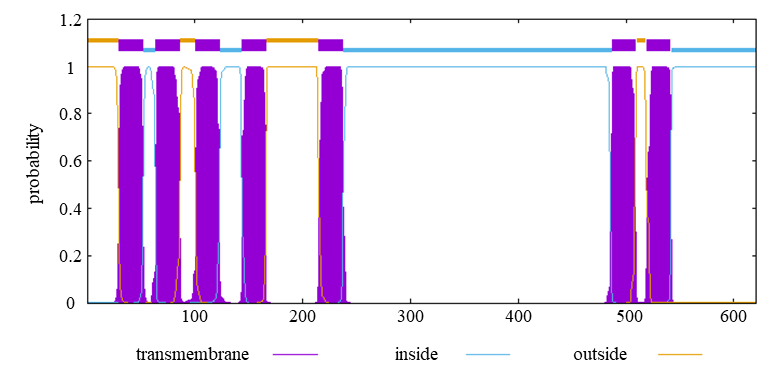
Supplementary Figure S3.**

**Supplementary Figure S3.** Hydropathy profile of the predicted RpOAα1-R amino acid sequence. The hydropathy profile was obtained using the TMHMM server v. 2.0 server. Amino acids numbers are given on the axis and the transmembrane segments are represented.
