# Supplementary material for: The octopamine receptor OAα1 influences oogenesis and reproductive performance in Rhodnius prolixus
Source: PLoS One. 2023 Dec 29;18(12):e0296463. doi: 10.1371/journal.pone.0296463 (PMC10756544; doi:10.1371/journal.pone.0296463)
Supplement: S3 Fig — (DOCX) [file pone.0296463.s003.docx]

**Supplementary Figure S4.**

**
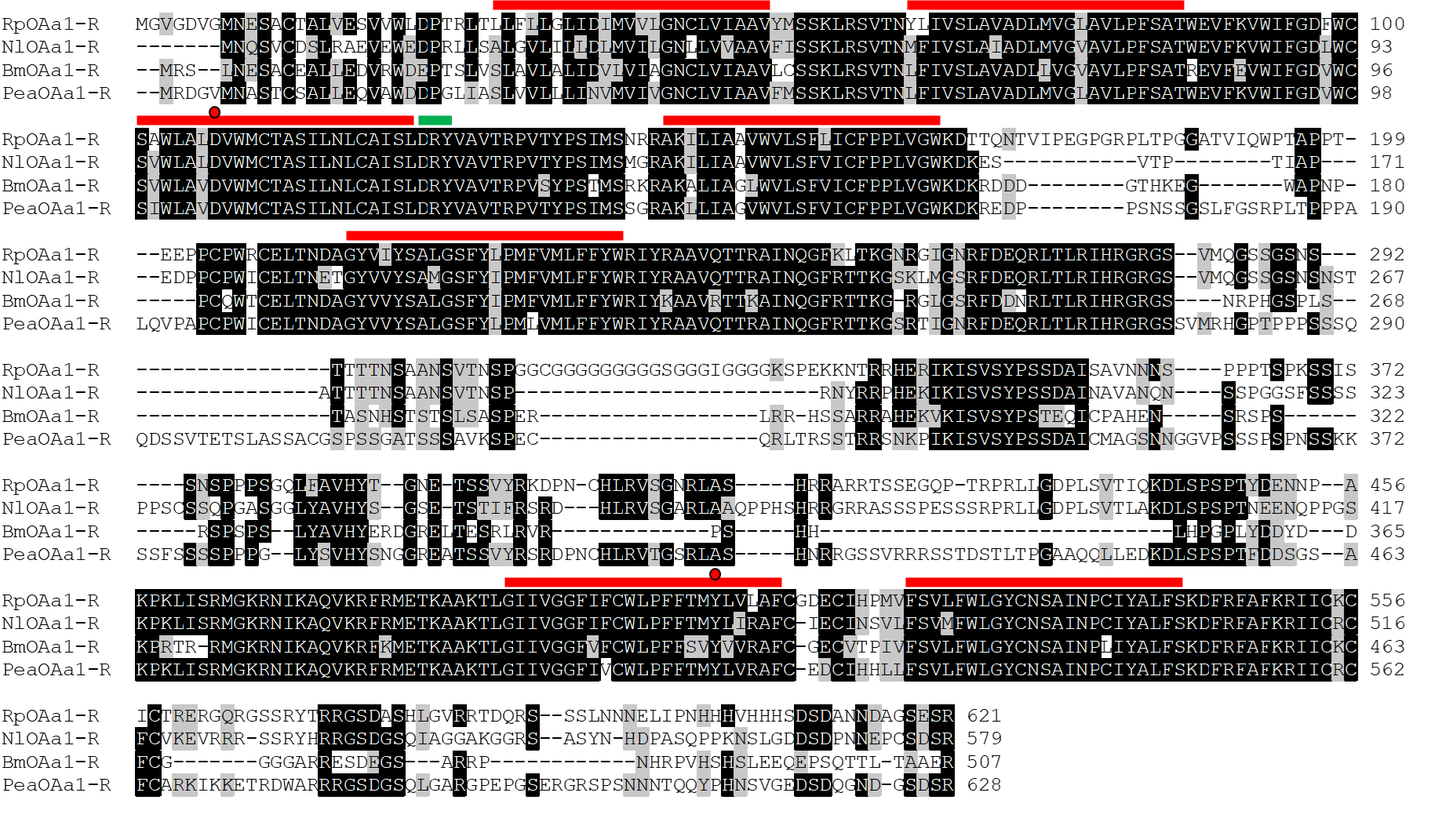
**

**Supplementary Figure S4**. Amino acid sequence alignment of RpOAα1-R with orthologous receptors from *Nivaparlata lugens* (NlOAα1-R), *Bombyx mori* (BmOAα1-R) and *Periplaneta americana* (PeaOAα1-R). The putative seven transmembrane domains are indicated by red lines. Identical residues are highlighted in black while conservative substitutions are in grey. A red circle indicates the conserved aspartic acid and the phenylalanine residues that could interact with OA. The DRY domain is highlighted by a green line.
