# Supplementary material for: The octopamine receptor OAα1 influences oogenesis and reproductive performance in Rhodnius prolixus
Source: PLoS One. 2023 Dec 29;18(12):e0296463. doi: 10.1371/journal.pone.0296463 (PMC10756544; doi:10.1371/journal.pone.0296463)
Supplement: S4 Fig — (DOCX) [file pone.0296463.s004.docx]

**Supplementary Figure S5.**

**
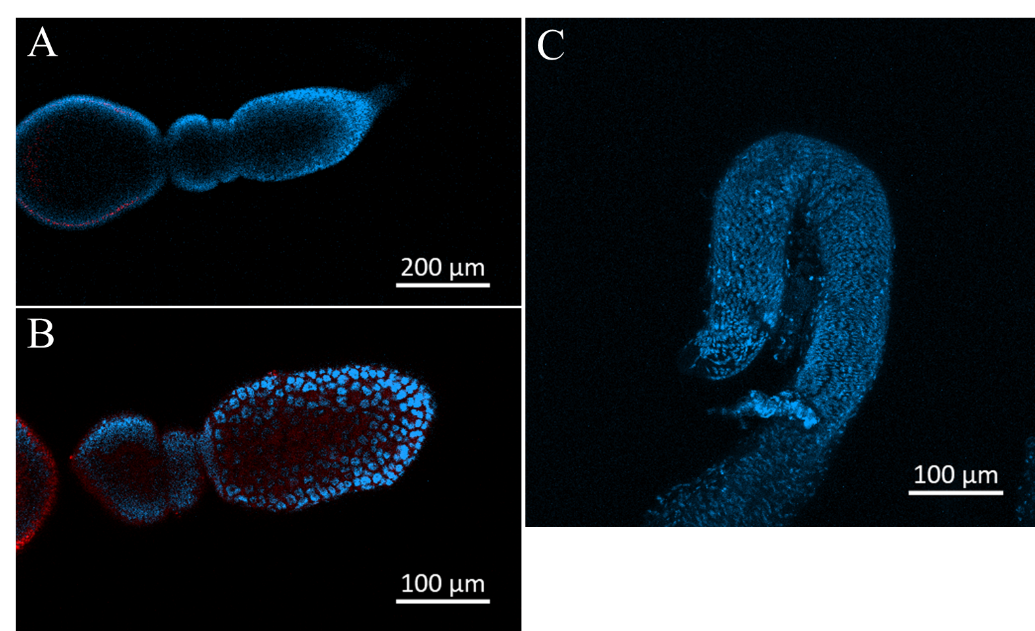
**

**Supplementary Figure S5.** Controls for fluorescent in situ hybridization of *RpOAα1-R* transcript in the reproductive system of *R. prolixus* adult females. The antisense RNA probe was used on tissues from *R. prolixus* adult females injected with dsOAα1. Lower or no signal was present across the ovariole (**panel A**), the tropharium (**panel B**), and the spermatheca (**panel C**). The images were acquired using a Zeiss laser scanning confocal microscope LSM510 and the LSM image browser software (Carl Zeiss, Jena, Germany). FISH experiments were repeated twice, using 3 reproductive systems in each experiment. Nuclei were counterstained with DAPI (blue)
