# Supplementary material for: The octopamine receptor OAα1 influences oogenesis and reproductive performance in Rhodnius prolixus
Source: PLoS One. 2023 Dec 29;18(12):e0296463. doi: 10.1371/journal.pone.0296463 (PMC10756544; doi:10.1371/journal.pone.0296463)
Supplement: S5 Fig — (DOCX) [file pone.0296463.s005.docx]

**Supplementary Figure S6.**


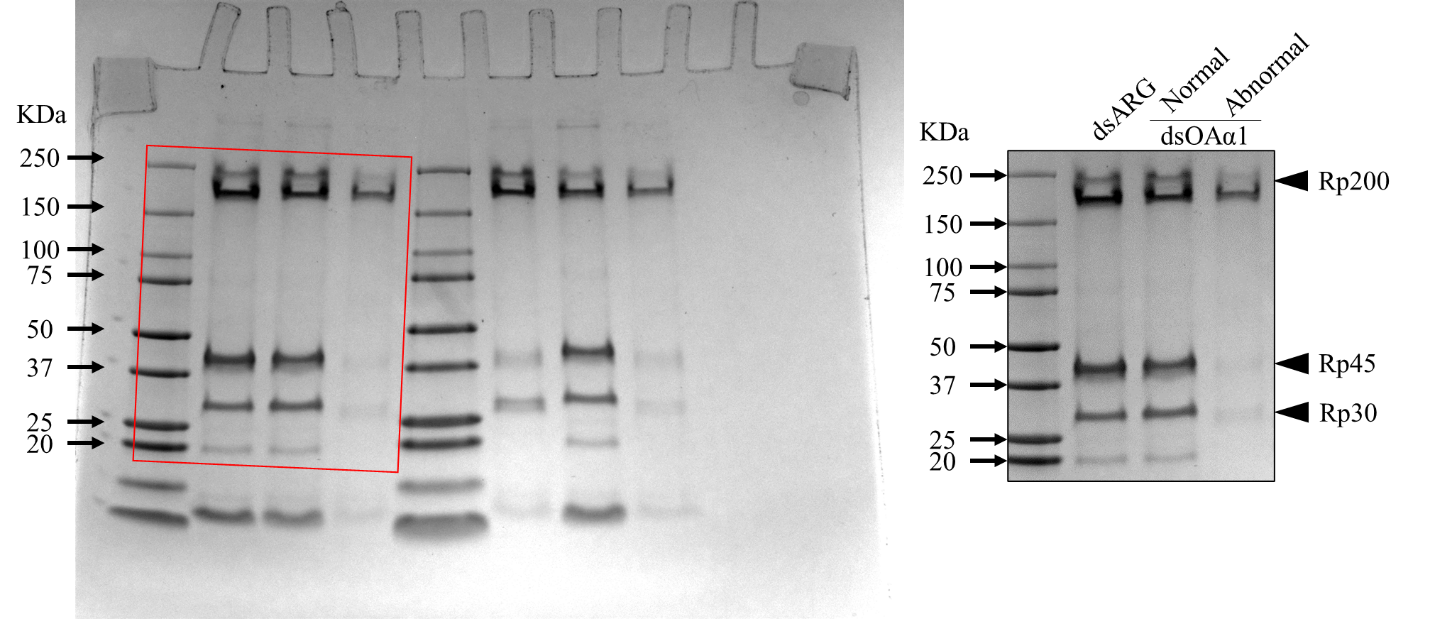


**Supplementary figure S6.** A representative uncropped image of a gel of chorion proteins as shown in **Figure 5, panel D**, shown on the right. Protein extracts from egg homogenates of insects injected with dsRNAs, were subjected to pre-made gels (4-20 % Mini-PROTEAN™ TGX Stain-Free™ Protein Gels, 10 well, 30 µl) to detect chorion proteins.
