# Supplementary material for: The octopamine receptor OAα1 influences oogenesis and reproductive performance in Rhodnius prolixus
Source: PLoS One. 2023 Dec 29;18(12):e0296463. doi: 10.1371/journal.pone.0296463 (PMC10756544; doi:10.1371/journal.pone.0296463)
Supplement: S1 Table — (DOCX) [file pone.0296463.s007.docx]

**Supplementary table T2.** Molecular features of *R. prolixus* OAα1-R.

| **Potential N-glycosylation sites** | **Potential P-sites (PKA)** | **Potential P-sites (PKC)** |
| --- | --- | --- |
| N^9^ | T^29^; S^345^ | S^55^; S^56^; T^130^; T^189^; T^246^; T^257^; T^287^; T^296^; T^365^; S^437^ |
