# Supplementary material for: The octopamine receptor OAα1 influences oogenesis and reproductive performance in Rhodnius prolixus
Source: PLoS One. 2023 Dec 29;18(12):e0296463. doi: 10.1371/journal.pone.0296463 (PMC10756544; doi:10.1371/journal.pone.0296463)
Supplement: S3 Table — (DOCX) [file pone.0296463.s009.docx]

**Supplementary Figure S1.** *R. prolixus* OAα1-R sequence: regions chosen for dsRNA are highlighted.

**ATG**GGTGTTGGTGACGTAGGTATGAACGAATCAGCGTGTACAGCTTTAGTTGAATCAGTAGTGTGGTTGGATCCGACTCGTCTAACATTGTTGTTCCTATTAGGGCTAATCGACATTATGGTCGTTTTAGGTAATTGCCTAGTGATTGCCGCTGTATATATGTCTTCTAAACTGCGATCGGTTACCAATTATCTTATTGTATCGTTAGCTGTAGCCGATCTGATGGTCGGACTTGCTGTACTTCCGTTCAGCGCCACATGGGAAGTGTTCAAGGTTTGGATATTCGGGGACTTCTGGTGTTCAGCGTGGCTAGCGTTAGACGTCTGGATGTGCACAGCATCAATACTCAATCTTTGCGCTATCTCCTTGGACAGGTATGTGGCCGTCACAAGACCAGTCACATATCCTAGTATTATGTCGAACAGGCGAGCTAAGATACTCATAGCGGCCGTATGGGTGTTGTCGTTTTTGATTTGTTTTCCGCCGTTGGTCGGATGGAAGGACACTACGCAAAATACCGTCATACCAGAAGGACCAGGCAGGCCGTTGACACCTGGAGGCGCTACCGTCATACAATGGCCGACAGCACCGCCCACCGAGGAACCACCCTGTCCTTGGCGTTGCGAGTTGACCAACGACGCTGGTTATGTAATCTATTCAGCGTTGGGCAGCTTCTATCTGCCCATGTTTGTGATGTTGTTCTTTTATTGGAGAATCTATAGAGCGGCCGTGCAAACTACTAGAGCTATTAATCAAGGCTTCAAGTTAACCAAAGGGAATAGAGGTATTGGTAATAGATTTGATGAACAACGATTAACGCTGAGAATTCATCGTGGCCGTGGATCTGTGATGCAAGGGAGTAGTGGTAGTAACAGCACCACGACAACCAACTCGGCGGCTAATTCCGTCACTAATTCCCCAGGTGGTTGCGGCGGTGGTGGAGGAGGAGGAGGAGGAAGCGGAGGCGGAATTGGTGGCGGCGGCAAGTCACCCGAGAAGAAGAACACAAGACGCCATGAAAGGATCAAGATCAGCGTAAGTTATCCAAGCTCGGACGCTATTTCAGCGGTGAACAACAATTCTCCGCCACCAACAAGTCCAAAATCGTCGATATCCAGTAATTCACCGCCTCCAAGCGGCCAGCTATTCGCTGTACACTATACGGGTAATGAGACTAGCTCTGTATATAGAAAAGATCCGAATTGCCATTTACGGGTCAGCGGTAACCGTTTAGCATCGCATCGCCGAGCAAGGCGAACGAGCAGCGAAGGCCAACCGACCAGACCTAGGCTGTTGGGCGATCCTTTGAGCGTCACCATCCAGAAGGATCTCTCTCCGTCCCCGACCTACGATGAAAACAATCCAGCAAAACCGAAACTCATTTCCCGTATGGGCAAGAGAAACATAAAAGCCCAAGTGAAACGTTTTCGTATGGAGACCAAAGCGGCTAAAACGCTTGGCATAATCGTCGGCGGATTTATATTCTGTTGGCTCCCGTTCTTCACGATGTACTTAGTTTTAGCATTCTGCGGCGATGAGTGCATCCACCCGATGGTATTCTCAGTGCTATTCTGGCTGGGCTACTGTAATTCAGCCATAAATCCATGTATATACGCGCTGTTCAGCAAGGACTTCCGTTTCGCTTTTAAAAGAATCATCTGCAAATGTATATGCACACGAGAACGAGGCCAGCGCGGTTCGTCTAGGTATACTAGACGTGGTTCTGACGCTTCACATCTGGGCGTACGTAGAACGGATCAACGAAGCTCCTCGTTGAACAACAACGAACTCATACCGAATCACCATCACGTGCATCATCACTCAGACAGTGATGCGAACAATGATGCCGGCTCAGAGTCCAGG**TGA**

MGVGDVGMNESACTALVESVVWLDPTRLTLLFLLGLIDIMVVLGNCLVIAAVYMSSKLRSVTNYLIVSLAVADLMVGLAVLPFSATWEVFKVWIFGDFWCSAWLALDVWMCTASILNLCAISLDRYVAVTRPVTYPSIMSNRRAKILIAAVWVLSFLICFPPLVGWKDTTQNTVIPEGPGRPLTPGGATVIQWPTAPPTEEPPCPWRCELTNDAGYVIYSALGSFYLPMFVMLFFYWRIYRAAVQTTRAINQGFKLTKGNRGIGNRFDEQRLTLRIHRGRGSVMQGSSGSNSTTTTNSAANSVTNSPGGCGGGGGGGGGSGGGIGGGGKSPEKKNTRRHERIKISVSYPSSDAISAVNNNSPPPTSPKSSISSNSPPPSGQLFAVHYTGNETSSVYRKDPNCHLRVSGNRLASHRRARRTSSEGQPTRPRLLGDPLSVTIQKDLSPSPTYDENNPAKPKLISRMGKRNIKAQVKRFRMETKAAKTLGIIVGGFIFCWLPFFTMYLVLAFCGDECIHPMVFSVLFWLGYCNSAINPCIYALFSKDFRFAFKRIICKCICTRERGQRGSSRYTRRGSDASHLGVRRTDQRSSSLNNNELIPNHHHVHHHSDSDANNDAGSESR

**Supplementary Figure S1**: RpOAα1-R sequence is shown with the positions of the primers used for dsRNA synthesis, RT-qPCR analysis, and cloning. The start codon ATG is highlighted in green while the stop codon TGA is labeled in red. In total, the RpOAα1-R sequence results in 1866 nucleotide longs. The resulting dsRNA fragments (409 bp long) used to knock down RpOAα1-R transcripts is highlighted in yellow. The primers used are underlined.
